# Enhancing Methane Production in Anaerobic Digestion of Food Waste Using Co-Pyrolysis Biochar Derived from Digestate and Rice Straw

**DOI:** 10.3390/molecules30081766

**Published:** 2025-04-15

**Authors:** Qinyan Yang, Huanran Liu, Li Liu, Zhen Yan, Chunmeng Chui, Niannian Yang, Chen Wang, Guoqing Shen, Qincheng Chen

**Affiliations:** 1 School of Agriculture and Biology, Shanghai Jiao Tong University, Shanghai 200240, China; kensi_0812@sjtu.edu.cn (Q.Y.); sjtu_lhr@sjtu.edu.cn (H.L.); liul97@sjtu.edu.cn (L.L.); chenwang@sjtu.edu.cn (C.W.); 2 Shanghai Pudong Development (Group) Co., Ltd., Shanghai 200127, China; timothyyan@126.com; 3 Shanghai Liming Resources Reuse Co., Ltd., Shanghai 201209, China; 18700742813@163.com (C.C.); yangnn1025@163.com (N.Y.); 4 Shanghai Yangtze River Delta Eco-Environmental Change and Management Observation and Research Station (Shanghai Urban Ecosystem Research Station), Ministry of Science and Technology, National Forestry and Grassland Administration, 800 Dongchuan Rd., Shanghai 200240, China

**Keywords:** anaerobic digestion, biochar, co-pyrolysis, waste recovery, methane production

## Abstract

Anaerobic digestion (AD) is a preferred method for food waste (FW) treatment due to its sustainability and potential for production of renewable bioenergy. However, the accumulation of volatile fatty acids (VFAs) and ammonia often destabilizes the AD process, and managing the digestate byproduct poses additional challenges. This study investigates the use of co-pyrolysis biochar synthesized from digestate and rice straw (DRB) to enhance methane production and AD efficiency. DRB addition increased cumulative methane yield by 37.1%, improved VFA conversion efficiency, and achieved a 42.3% higher NH_3_-N-removal rate compared to the control group. The COD-removal rate was 68.7% throughout the process. Microbial analysis revealed that DRB selectively enriched *Fastidiosipila* and *Methanosarcina*, promoting direct interspecies electron transfer (DIET) and methane yield. These findings highlight DRB’s potential to enhance AD efficiency and support closed-loop resource utilization.

## 1. Introduction

The rapid growth in the global population has significantly increased the generation of food waste (FW) [1,2], which is projected to rise by 20–68% by 2050 [3]. The complex composition, high moisture content, and substantial organic-matter content of FW pose significant challenges for its treatment and conversion [4]. Currently, most FW is managed through unsustainable methods such as landfilling and incineration [5], contributing to various environmental issues [6,7]. Therefore, there is an urgent need for treatment technologies that enable efficient resource recycling and utilization.

Anaerobic digestion (AD) has garnered widespread attention due to its multiple advantages, including low secondary pollution [8,9], high biosafety [10], cost-effectiveness [11,12], and the production of renewable bioenergy in the form of methane [13]. AD is a biological process in which microorganisms degrade organic substrates under anaerobic conditions, ultimately converting them into methane [14]. The process comprises several biochemical stages, typically categorized as hydrolysis, acidogenesis, acetogenesis, and methanogenesis [15,16,17]. However, AD is constrained by several factors, including the accumulation of volatile fatty acids (VFAs) [18] and system instability caused by excessive ammonia nitrogen levels [19]. Furthermore, AD generates by-products such as biogas slurry and digestate [20,21]. Among these, digestate—comprising both solid and liquid fractions—poses the greatest disposal challenge due to its high organic-matter content, which further complicates resource-recovery efforts [5]. Enhancing methane production while utilizing digestate efficiently is critical for improving AD systems.

Previous studies have demonstrated that methane production and stability in the AD process [22,23] can be improved by adding biochar (BC) [24,25,26], a carbon-rich material produced through biomass pyrolysis under limited oxygen conditions [27,28]. For instance, Wang et al. [29] determined that Fe_2_O_3_-modified biochar derived from digestate increased the methane yield of kitchen waste by 20.8%, improving pH stability by adsorbing VFAs and mitigating acidification. Similarly, Liu et al. [30] reported that adding biochar derived from biogas residue enhanced the anaerobic co-digestion of sewage sludge and food waste, improving methane production by 22.6%. BC facilitates direct interspecies electron transfer (DIET), supplies trace elements, and provides porous structures for microbial colonization [19,30,31]. However, BC properties vary significantly based on pyrolysis conditions and feedstocks, limiting its effectiveness in AD [32]. For example, rice straw has a relatively high C/N ratio and a very low moisture content, whereas digestate is characterized by a rich diversity of microbes, along with high contents of N, P, and organic matter [33,34].

Co-pyrolysis technologies address these limitations by combining multiple feedstocks [35], enhancing BC characteristics and facilitating waste reduction [36,37]. The process contributes to waste reduction by employing various mixed-waste feedstocks [38,39]. Co-pyrolysis BC has shown promise in pollutant adsorption [40], soil remediation [41], and waste valorization [35]. However, its potential to enhance AD remains underexplored [42].

This study examines the use of co-pyrolysis biochar derived from digestate and rice straw to enhance methane production during AD. By analyzing the physicochemical properties of different biochars, this work provides insights into DRB’s role in improving methane production and AD efficiency, promoting sustainable, closed-loop resource utilization.

## 2. Results and Discussion

### 2.1. Comparison of the Characteristics of Materials

The results for specific surface area (Table 1) highlight differences among DB, RB, and DRB, emphasizing the role of biochar’s porous structure in providing a habitat for microbial growth [43]. The N_2_ adsorption–desorption isotherms (Figure S3) of DRB materials exhibited Type-I behavior, with H4 hysteresis loops at higher pressure (P/P_0_ = 0.5–1.0), indicating the presence of mesopores. Mesopores (2–50 nm) provide abundant contact sites, whereas smaller pores are more susceptible to blockage by other particles, leading to reduced ammonium adsorption [44]. Although DRB does not possess the highest specific surface area among the three materials, its mesoporous structure significantly enhances ammonium adsorption. Electrical conductivity (EC) is another critical factor influencing DIET [45,46]. The EC values of DB, RB, and DRB were measured at 4.48, 4.56, and 4.88 mS/cm, respectively, with DRB exhibiting the highest conductivity, which may enhance the DIET process.

SEM images of DB, RB, and DRB (Figure S1) revealed that all biochar types exhibited a fluffy surface with pronounced porous structures. The rapid decomposition of organic components during low-temperature pyrolysis released gases such as CO and CO_2_, forming larger pores [47]. Notably, DRB displayed a regular and delicate surface with prominent stomata and lamellar structures, resulting in reduced ash accumulation within its pores compared to those of DB and RB. Conversely, DB and RB exhibited particulate deposits on their surfaces due to the encapsulation of non-volatile ash by carbonization products [48].

XPS analysis (Figure S2) quantified the surface chemical composition and elemental states of the biochar. The C_1s_ contents were 53.4% in DB, 77.6% in RB, and 66.0% in DRB, while O_1s_ contents were 42.9%, 21.0%, and 33.4%, respectively. During pyrolysis, raw materials undergo oxidation processes, converting C-C and C=O bonds into C-O bonds, which results in a higher proportion of C-O bonds in DB, RB, and DRB. The C-O/C=O ratio in DRB (2.64) significantly exceeded those in DB and RB, likely due to the alkali in the digestate neutralizing hydroxyl groups [49]. C-O functional groups, such as phenolic hydroxyls, are reductive and enhance electron-donating capacity [50]. In contrast, C=O groups, associated with oxidizing entities like carbonyl and quinone structures, act as electron acceptors [51]. DRB’s higher C-O/C=O ratio indicates a better balance between reductive and oxidizing groups, enhancing electron-transfer capacity. This property likely supports DIET in AD, boosting microbial activity and methane production.

FTIR spectroscopy (Figure S2b) was used to further analyze the surface functional groups of the biochar. All biochar types displayed common characteristic peaks, including the stretching vibration of intermolecular -OH groups (3436 cm^−1^), C=C vibrations (1619 cm^−1^, 1637 cm^−1^), and C-H bending vibrations (1350 cm^−1^, 1384 cm^−1^). Notably, the C=C peak at 1619 cm^−1^ in DRB corresponds to carbon–carbon double bonds in aromatic rings, which can be part of quinone structures. Quinones, characterized by a C=O group attached to a double bond, form during pyrolysis and enhance biochar’s electron-transfer capacity and surface reactivity [52]. The distinct quinone C=C peak in DRB suggests that co-pyrolysis facilitated the formation of reactive quinone-like compounds, further improving its reactivity and electron-transfer properties.

The thermal stability of the biochar was assessed using thermogravimetric analysis (Figure S2c). Significant weight loss was seen in RB at 380–700 °C, in DB at 800–900 °C, and in DRB at 760–900 °C, with DRB showing less severe weight loss compared to DB and RB. Residual masses were 69.74% for DB, 69.54% for RB, and 72.5% for DRB, indicating that DRB had the highest thermal stability among the three biochar types. Co-pyrolysis involves the simultaneous thermal decomposition of multiple feedstocks within a single system [53,54]. Unlike the simple combination of individual biochars, the resulting product reflects synergistic interactions between the feedstocks [54]. DRB exhibits a more complex structure, characterized by higher aromaticity, which may enhance its resistance to decomposition [55]. XPS analysis (Figure S2d–i) reveals that DRB has the lowest C=O content, likely due to high-temperature chemical interactions between straw and biogas residue. This structural modification contributes to its improved thermal stability [56].

### 2.2. Regulation of DB, RB, and DRB on Anaerobic Digestion of Food Waste

#### 2.2.1. Impact on Yields of Biogas and Methane 

**Figure 1:**
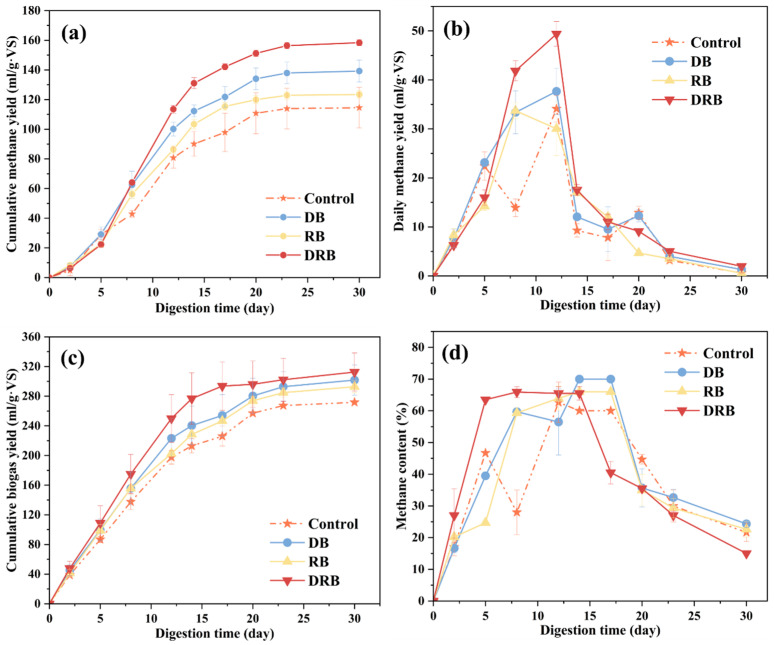
Methane production by different groups: (**a**) cumulative methane yield; (**b**) daily methane yield; (**c**) cumulative biogas yield; (**d**) methane content. Data for each group were acquired from three parallel sets of tests.

Biogas production across groups with different material additions is illustrated in Figure 1. The addition of biochar (BC) significantly enhanced cumulative methane production in all treatment groups compared to the control (*p* < 0.05). By day 23 of AD, the DRB group achieved the highest cumulative methane yield, at 156 (±7.01) mL/(g·VS), outperforming the DB, RB, and control groups, which produced 138 (±8.23), 123 (±5.17), and 114 (±11.25) mL/(g·VS), respectively (Figure 1a). Notably, DRB addition increased total methane production by 37% relative to the control. At the conclusion of AD, based on the total production (Table S2), methane production in the control, DB, RB, and DRB groups reached 114.56 (±16.02), 139.25 (±17.20), 123.47 (±7.13), and 158.38 (±5.07) mL/(g·VS), respectively (Figure 1a). A similar trend was observed in total biogas production (Figure 1c), with yields of 271.82 (±6.00), 301.70 (±20.02), 292.73 (±12.10), and 312.50 (±8.07) mL/(g·VS) for the control, DB, RB, and DRB groups, respectively.

Daily methane yields for most groups displayed two distinct peaks, although their durations varied (Figure 1b). The first peak, occurring from day 8 to day 12, saw the DRB group reach a maximum yield of 49 (±10.11) mL/(g·VS), while the RB group recorded the lowest peak of 30 (±9.20) mL/(g·VS). The variations in the second peak, occurring between days 17 and 23, further emphasized the variability in methane-production dynamics among the groups.

#### 2.2.2. Variations in pH and Volatile Fatty Acids

**Figure 2:**
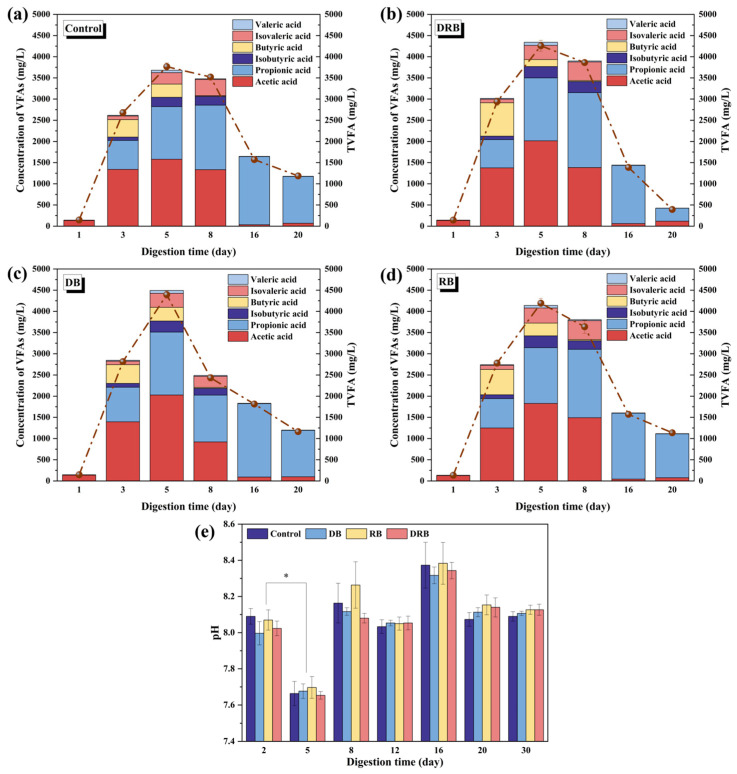
Changes in VFA concentrations in the AD system of food waste: (**a**–**d**) VFA composition in experimental groups; (**e**) pH variations. Statistically difference between day 2 and 5: *p* < 0.05 (*). Data were collected from three parallel experimental sets.

The changes in VFAs and pH during the AD process are shown in Figure 2. VFAs, critical intermediates in AD, include acetic acid, propionic acid, butyric acid, and valeric acid. Imbalances between VFA production and consumption can destabilize the AD process [57]. At the start of AD (Figure 2a–d), concentrations of total volatile fatty acids (TVFAs) in all systems were below 500 mg/L, with acetic acid predominating. By day 5, all groups reached their peak TVFA concentrations. The DRB group exhibited a peak TVFA concentration of 4341 (±3.03) mg/L, which exceeded the control group’s peak (3680 (±19.34) mg/L) by 22.1%, demonstrating DRB’s superior ability to enhance acid production in AD systems (Figure 2a–d). After peaking, TVFAs rapidly decreased, primarily due to a marked reduction in acetic acid concentration. During this phase, all treatment groups showed significantly greater reductions in acetic acid compared to the control group, indicating that BC addition notably promotes VFA consumption during AD.

In the initial five days of AD, pH values (Figure 2e) across all groups dropped below 7.60 as VFAs accumulated rapidly. The addition of BC initially caused a slight pH increase. However, from the start of AD to day 5, a significant pH decline was observed in all groups (*p* < 0.05), consistent with the accumulation of VFAs. By day 8, pH levels in all groups gradually increased to approximately 8. Beyond day 8, pH levels stabilized across all groups, aligning with findings from previous studies [58], which identified an optimal pH range of 7.00–7.50 for methanogen activity.

#### 2.2.3. Ammonia Nitrogen and COD

**Figure 3:**
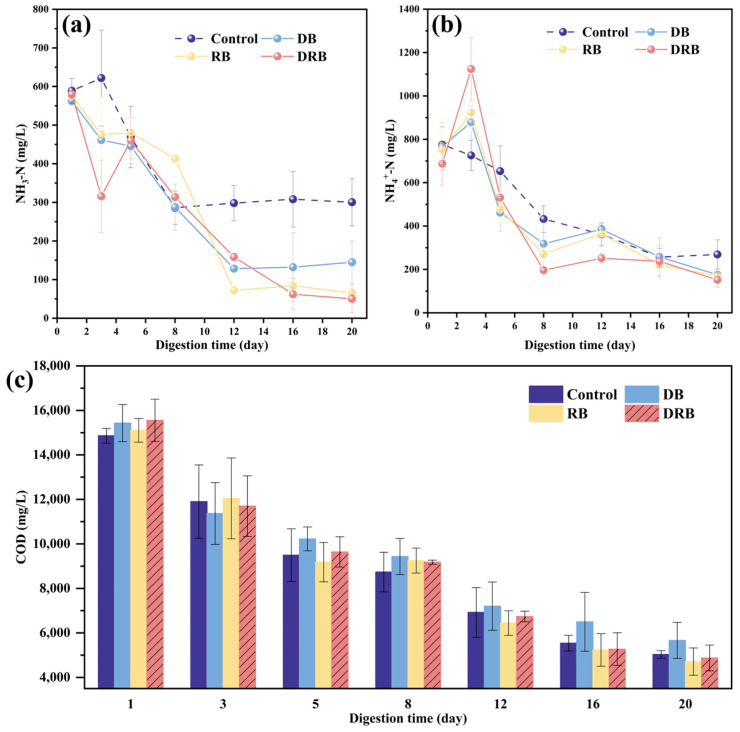
Changes in NH_3_-N concentrations in the AD system of FW (**a**); changes in NH_4_^+^-N concentrations in the AD system of FW (**b**); changes in COD in the AD system of FW (**c**).

High ammonia nitrogen concentrations can inhibit methane production by reducing the abundance and activity of methane-related genes and enzymes, ultimately lowering AD efficiency [19]. Ammonia nitrogen in water typically exists as ionized ammonium (NH_4_^+^-N) and unionized ammonia (NH_3_-N) [59,60]. In this study, the NH_3_-N removal rate for the control group was 49.01%, while the experimental groups—DB, RB, and DRB—achieved significantly higher removal rates of 74.21%, 88.66%, and 91.32%, respectively (Figure 3a). The NH_3_-N removal rate in the control group was significantly lower than those in all experimental groups (DB, RB, and DRB). Additionally, the removal rate in the DB group was significantly lower than those of the RB and DRB groups. However, no significant difference was observed between the RB and DRB groups (*p* > 0.05), indicating comparable performance between the two. Among the experimental groups, DRB demonstrated exceptional performance, achieving a 42.31% improvement over the control group. As shown in Figure 3b, the concentration of NH_4_^+^-N increased in all treatments except for the control group on day 3, suggesting that the addition of BC facilitated the hydrolysis rate of AD, thereby promoting the release of substantial amounts of NH_4_^+^. By day 20, the greatest decrease in NH_4_^+^-N concentration was observed in the DRB. Overall, the highest removal of ammonia nitrogen by DRB may be attributed to the protonation of functional groups (C=O, COO^−^) on DRB surfaces (Figure S2d–i), which imparts a partial positive charge, thereby reducing the polar attraction of NH_4_^+^ ions in solution [61] and contributing to pH regulation. Similar adsorption mechanisms have been reported in studies utilizing BC and zeolite for ammonia nitrogen removal [62,63,64].

Effective COD degradation, a key indicator of AD performance, enhances the breakdown of organic matter and boosts biogas production [65]. During the first biogas-production peak (day 3), COD levels across all treatment groups decreased rapidly (Figure 3c). COD-removal rates for the control, DB, RB, and DRB groups were 19.91%, 26.34%, 20.24%, and 24.79%, respectively. The addition of biochar (DB, RB, and DRB groups) enhanced initial COD-removal rates, a change correlated with increased daily methane production during this phase. By the end of fermentation, the COD removal rates reached 66.13%, 63.29%, 68.80%, and 68.66% for the control, DB, RB, and DRB groups, respectively, highlighting the potential of DRB to enhance the efficiency of COD removal.

### 2.3. Microbial Diversity Analysis

Microbial community structure and diversity were analyzed for bacteria and archaea on days 5 and 16 in AD systems supplemented with DB, RB, and DRB. A Venn diagram (Figure S4) depicts the similarities and overlapping proportions of microbial communities among the samples. On day 5, a total of 591 bacterial OTUs were identified across all samples, with this number increasing to 665 by day 16. This increase reflects shifts in the microbial community driven by the various supplementing materials. Similarly, the number of shared archaea exhibited slight fluctuations between days 114 and 121, as well as between days 5 and 16.

#### 2.3.1. Bacterial Community Analysis

**Figure 4:**
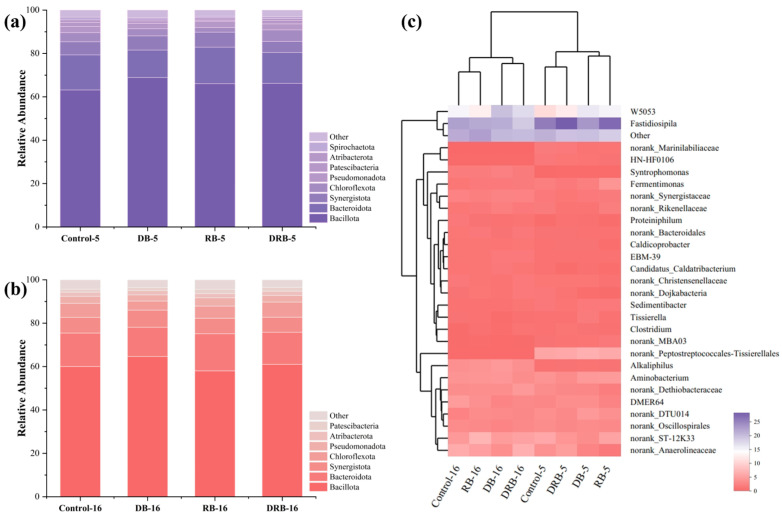
Bacterial community structures at the phylum level on day 5 (**a**) and day 16 (**b**); genus level on day 5 and day 16 (**c**).

Figure 4a,b shows that the phylum Bacillota, which is involved in the hydrolysis and acidification of organic macromolecules, was the dominant bacterial group. The competitive advantage of Bacillota in the high-VFA environment of the reactor can be attributed to its ability to form spores under harsh conditions [66]. Despite the addition of DB, RB, and DRB, Bacillota consistently remained the most abundant group (~62.85%), indicating that these materials had limited influence on the restructuring of bacterial communities.

At the genus level, 21 bacterial sequences with relative abundances exceeding 1% were identified on day 5 (Figure 4c). The dominant genera included *Fastidiosipila*, *W5053*, *Aminobacterium*, *DMER64*, *Fermentimonas*, and *Syntrophomonas*. The relative abundances of one of these genera, *Fastidiosipila*, in the DB, RB, and DRB groups were 21.6%, 25.2%, and 26.1%, respectively. The high abundance of *Fastidiosipila* in the DRB group can be attributed to its critical role in hydrolysis and acidification; it converts complex organic macromolecules into VFAs and CO_2_ [67].

The genus *W5053*, belonging to Bacillota, ranked second in abundance. It is integral to the acid-producing process, with acetate as its main metabolic product [68]. *W5053* also interacts synergistically with *Methanothrix* [69,70], suggesting that its higher abundance in the DRB group facilitated VFA generation and subsequent methane production. Additionally, *DMER64* was more prevalent in DRB reactors, identifying it as a syntrophic butyrate-oxidizing bacterium capable of establishing DIET through pore-mediated mechanisms [71]. The data suggest that DRB supplementation enhanced DIET by facilitating electron transfer in *DMER64* through mechanisms that substitute conductive pili [72].

#### 2.3.2. Archaeal Community Analysis

**Figure 5:**
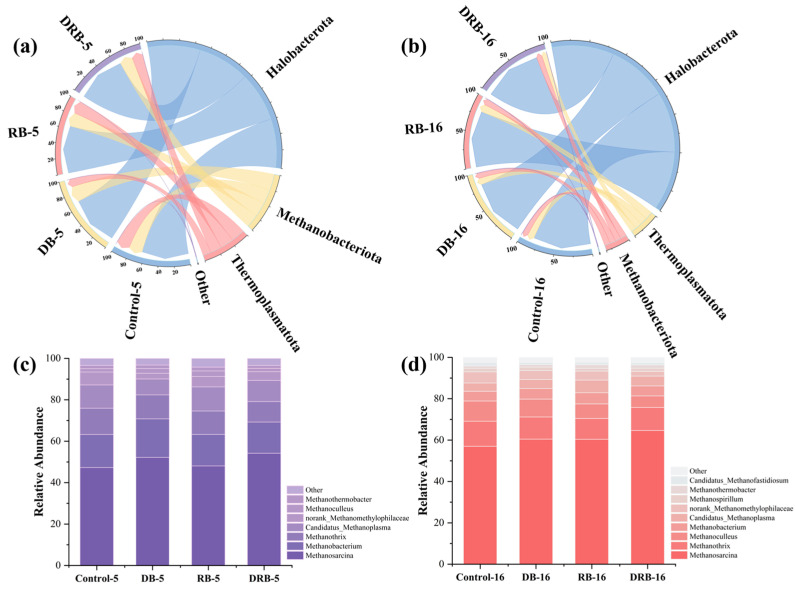
Archaeal community structure at the phylum level on day 5 (**a**) and day 16 (**b**); genus-level analysis on day 5 (**c**) and day 16 (**d**).

Changes in the archaeal community during AD were analyzed, and the results are presented in Figure 5. At the phylum level (Figure 5a,b), dominant taxa across all groups included Halobacterota, Methanobacteriota, and Thermoplasmatota. In the control group, the abundance of Halobacterota increased markedly from 63.3% on day 5 to 82.1% on day 16. This rise reflects the archaea’s adaptation to stress associated with elevated VFAs in FW, suggesting that high VFA concentrations suppressed methane yield in the control group.

At the genus level (Figure 5c,d), seven dominant Archaea were identified: *Methanosarcina*, *Methanobacterium*, *Methanothrix*, *Methanoculleus*, *Methanothermobacter*, and *Methanospirillum*. *Methanosarcina* predominated in all AD reactors and is capable of producing methane via the acetate, H_2_/CO_2_, and methyl organics pathways. Similarly, *Methanobacterium* is widely found in AD reactors, where it utilizes H_2_ and CO_2_ for methane production [29]. The coexistence of *Methanobacterium* and *Methanothrix* indicates the presence of both hydrogenotrophic and acetoclastic methanogenesis pathways [73].

**Figure 6:**
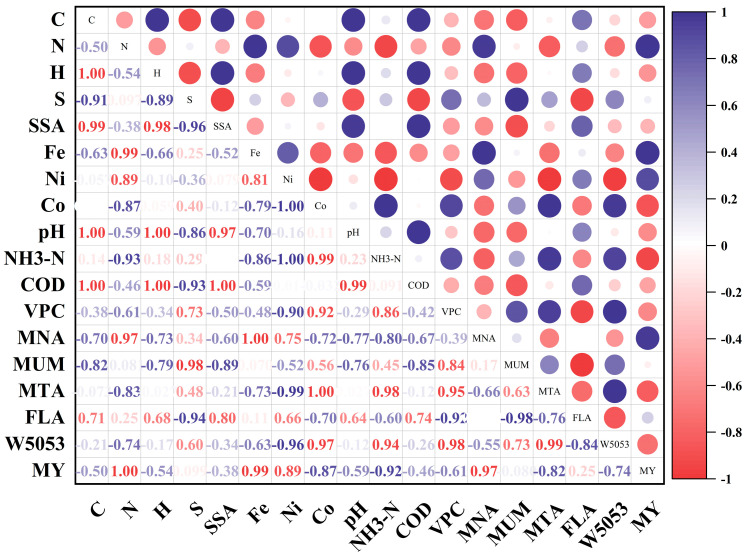
Heatmap of the Pearson correlation matrix for environmental factors. SSA: specific surface area; VPC: peak VFA concentration; MNA: *Methanosarcina* proportion at day 5; MUM: *Methanobacterium* proportion at day 5; MTA: *Methanothrix* proportion at day 5; FLA: *Fastidiosipila* proportion at day 5; W5053: *W5053* proportion at day 5; MY: methane yield.

On day 5, the relative abundance of *Methanosarcina* was 47.3% in the control group and was higher, at 52.2%, 48.1%, and 54.2%, in the DB, RB, and DRB groups, respectively. Prior research has shown that *Methanosarcina* is a mixotrophic methanogen capable of degrading acetic acid and facilitating the electron transfer essential for methane production through DIET [74]. Correlation analysis (Figure 6) confirmed a strong association between *Methanosarcina* abundance and methane yield, reinforcing the role of DRB in enhancing methane production via DIET and establishing methanogenic zones within the AD reactor.

Compared to individual BC treatments (DB and RB), DRB combined the regulatory benefits of both materials, significantly promoting the proliferation of *Methanosarcina*. By day 5, the relative abundance of *Methanosarcina* in the DRB group (54.2%) exceeded that in the control group (47.3%), and this advantage persisted through day 16. Interestingly, despite the superior methane yield in DRB reactors, the predominant genera did not dominate in relative abundance, suggesting that the enhanced methane yield arose from synergistic interactions among methanogens.

The co-occurrence of *Methanothrix* and *Methanobacterium* underscores the simultaneous contribution of hydrogenotrophic and acetoclastic methanogenesis to methane production. The increased abundance of *Methanobacterium* on day 5 suggests the activity of a dominant hydrogenotrophic pathway during FW AD. DRB enhanced microbial activity and methane production by leveraging the synergistic effects of DB and RB biochar. Microbial analysis further indicates that two key microorganisms were most abundant in the DRB group (Figure 4 and Figure 5), where they facilitated the DIET process. Direct interspecies electron transfer (DIET) between *Syntrophomonas* and *Methanothrix* enhances methane production and serves as a representative example of synergistic interactions driving DIET [75,76,77]. The observed increase in methane production can be attributed to three key effects of co-pyrolytic biochar. First, co-pyrolytic biochar facilitates DIET by providing functional groups and electrical conductivity that enable syntrophs to establish DIET chains, enhancing methane production [23,30]. Second, co-pyrolytic biochar contains trace elements such as Fe, Co, Ni, and Zn (Table 1), which stimulate enzyme synthesis, promote the growth of functional bacteria, and enhance hydrolytic acidification of substrate in the AD system [78,79]. Third, the porous structure offers ample attachment sites for microbes, accelerating microbial reproduction and fostering DIET relationships.

## 3. Materials and Methods

### 3.1. Materials

The FW sourced from Liming Resources Reuse Co., Ltd. (Shanghai, China) was household solid waste from residents in the Pudong District of Shanghai. The FW (68.40% water content) was composed of organic compounds (75.60%), i.e., carbohydrate (16.53%), protein (19.60%), and fat (9.53%). TS content in FW was determined to be 23.70%, while VS was measured at 21.15% (Table 1). The FW was pulverized and homogenized using a large industrial mill before being stored at −20 °C. The inoculum used was collected from an industrial-scale kitchen waste CSTR reactor in Liming Resources Reuse Co., Ltd. (Shanghai, China). The inoculum was adapted under anaerobic conditions at 37 °C for 7 days before it was used in the AD experiments. Before experimentation, incubation continued until biogas production became negligible. The TS, VS, and pH of the seed sludge were 2.29%, 1.90%, and 7.74, respectively. The detailed physicochemical properties of the FW and inoculum are summarized in Table 1.

Digestate and rice straw used for BC preparation were obtained from Liming Resources Organic Solid Waste (Shanghai, China). Total solids (TS) and volatile solids (VS) were measured in triplicate prior to drying the straw digestion solution, with a standard deviation of less than 0.05. The physicochemical properties of all materials are summarized in Table 1.

### 3.2. Biochar Preparation

Preparation of co-pyrolysis biochar from digestate and rice straw (DRB) was carried put as follows. Digestate and rice straw were dried at 105 °C until a constant weight was achieved. Fifty grams each of digestate and rice straw were coarsely ground in a grinder operating at 300 r/min and passed through a 50-mesh sieve to ensure uniform particle size. The homogenized mixture was transferred to a lidded porcelain crucible and pyrolyzed for 2 h at 600 °C in a muffle furnace, with a heating rate of 10 °C/min. After it had cooled overnight, the resulting powder was washed multiple times with deionized water, dried at 60 °C in an oven, and designated as DRB.

Preparation of digestate biochar (DB) and rice-straw biochar (RB) was carried put as follows. Digestate and rice straw were separately pyrolyzed under conditions identical to those used for DRB preparation.

### 3.3. Anaerobic Digestion Experimental Design and Set-Up

Anaerobic digestion was conducted in 500 mL serum bottles with a working volume of 400 mL. FW and inoculum were mixed at a 1:1 ratio on a VS basis, with an initial FW concentration of 8.8 g·VS/L. DB, RB, and DRB were added to experimental reactors at a concentration of 1.5 g/L. The experimental groups were added to reactors containing DB, RB, or DRB, while the reactor for the control group contained only FW and inoculum. A blank group, with a reactor containing only the inoculum, was also established. The biogas yields of both the control and experimental groups were adjusted by subtracting the background biogas production from that of the inoculum. After feeding, the reactors were purged with nitrogen (purity > 99.9%) for 5 min to create an anaerobic environment and placed in a 37 ± 2 °C constant-temperature shaker. Biogas was collected individually to monitor composition changes, and digestate samples were taken periodically. All experiments were performed in triplicate.

### 3.4. Analytical Methods

DB, RB, and DRB were degassed at 300 °C for 8 h before their adsorption–desorption isotherms were measured at 766 K using an automated physisorption analyzer (ASAP 2460, Micromeritics, Norcross, GA, USA). The MIRA3 scanning electron microscope (SEM) (TESCAN, Brno, Czech Republic) was used to analyze the surface morphology and composition of the gold-sprayed biochar samples. The samples were burned in a redox tube at 1200 °C and 850 °C, and their elemental compositions (N, C, H, S) were analyzed using an automatic elemental analyzer (Vario EL Cube, Elementar, GER). Functional groups were analyzed using Fourier-transform infrared spectroscopy (FTIR; Nicolet 6700, Thermo Scientific, Waltham, MA, USA) with a scanning range of 4000 to 500 c^−1^. Al/K target X-ray photoelectron spectroscopy (XPS; ESCALAB 250 Xi K-Alpha, Thermo Fisher, Waltham, MA, USA) was performed at an energy of 1480.6 eV, power of 150 W, and pass energy of 30 eV. The data were also corrected for binding energy using C_1s_ = 284.8 eV as a reference. The specific surface areas of the biochars were analyzed using the Brunauer–Emmett–Teller (BET) nitrogen adsorption–desorption method (ASAP 2460, Micromeritics, Norcross, GA, USA) at a degassing temperature of 300 °C. Particle-size distribution was determined using a laser particle size analyzer (Microtrac S3500, Microtrac, York, PA, USA). EC was assessed in a BC: water slurry at a ratio of 1:10 (*w*/*v*) using a conductivity meter (DDS-307 A, Shanghai INESA Scientific Instruments Co., Ltd., Shanghai, China).

Gas samples were collected using gas bags every two or three days and analyzed for biogas composition. The compositions of CH_4_, CO_2_, and H_2_ were analyzed using a GC-7890 A equipped with a thermal conductivity detector [21,80]. A 2 m × 3 mm column packed with TDX-01 (JK, Beijing, China) was used to separate different gas omponents in the gas sample. The carrier gas was argon.

TS, VS, pH, chemical oxygen demand (COD), and ammonia nitrogen (NH_3_-N and NH_4_^+^-N) were analyzed using standard methods [81,82]. NH_4_^+^-N measurements were conducted under constant temperature conditions. The digest of each group was centrifuged at 10,000 rpm for 8 min, and the supernatant was filtered through a 0.45 μm filter membrane. The filtrate collected was used to measure the physicochemical indexes. The volatile fatty acids (VFAs, C_2_–C_5_) were determined by gas chromatograph (GC-7000 D, Agilent Technology, Santa Clara, CA, USA) equipped with a flame ionization detector and a 30 m × 250 μm × 0.25 μm capillary column [83,84]. Nitrogen was used as the carrier gas. The temperatures of the initial oven, injection port, and detector were set to 100 °C, 145 °C, and 240 °C, respectively, with a temperature programming ramping from 100 °C to 240 °C at a rate of 5 °C/min up to 145 °C; this was followed by a rapid increase to 240 °C at 40 °C/min and an 8 min hold. The COD was measured using spectrophotometry-based test kits (Hach Lange LCK, Düsseldorf, Germany). NH_3_-N was detected using a salicylic acid method and spectrophotometry (DR5000, HACH, Loveland, CO, USA).

### 3.5. Analysis of Microbial Community

Digestate samples were collected during the stable gas-production phase of the acidification stage. Total DNA was extracted using a Fast DNA SPIN Soil Kit according to the manufacturer’s directions and analyzed by Sangon Biotech (Shanghai) Co., Ltd. (Shanghai, China). Bacterial and archaeal 16 S rRNA genes were amplified via PCR using the following primers: 341 F (50-CCTACGGGNGGCWGCAG-30)/805 R (50-GACTACHVGGGTATCTAATCC-30) and 349 F (50-GYGCASCAGKCGMGAAW-30)/806 R (50-GGACTACVSGGGTATCTAAT-30). The V3–V4 variable region was targeted for sequencing. Data analysis was performed using the Sangon Biotech platform. PCR products were extracted from 2% agarose gel, purified using the PCR Clean-Up Kit (YuHua, Shanghai, China) according to the manufacturer’s instructions, and quantified using a Qubit 4.0 fluorometer (Thermo Fisher Scientific, Waltham, MA, USA).

Purified amplicons were pooled in equimolar amounts and subjected to paired-end sequencing on an Illumina NextSeq 2000 platform (Illumina, San Diego, CA, USA) following the standard protocols of Majorbio Bio-Pharm Technology Co., Ltd. (Shanghai, China). Raw FASTQ files were demultiplexed using an in-house Perl script, quality-filtered with fastp v0.19.6 [85], and merged using FLASH v1.2.7 [86] under defined parameters. The optimized sequences were clustered into operational taxonomic units (OTUs) at 97% sequence similarity using UPARSE v7.1 [87,88], with the most abundant sequence in each OTU designated as the representative sequence. Taxonomic classification of OTU representative sequences was performed using RDP Classifier v2.2 [89] against the 16S rRNA gene database (e.g., Silva v138.2) with a confidence threshold of 0.7. Metagenomic functional predictions were conducted using PICRUSt2 (Phylogenetic Investigation of Communities by Reconstruction of Unobserved States) [90] based on OTU representative sequences.

### 3.6. Statistical Analysis

Experimental data were organized, analyzed, and visualized using Origin 2024. Variance analysis was performed with SPSS 20.0, considering *p*-values below 0.05 to be statistically significant. Paired *t*-tests were employed to compare biogas-production performance across groups. Standard deviations of multiple measurements were represented as error bars. Correlation analyses and data visualizations were conducted using R (4.4.1) (https://www.r-project.org/, accessed on 23 October 2024) with the cor and corrplot packages.

## 4. Conclusions

The co-pyrolysis of digestate with rice straw significantly enhanced the efficiency of FW AD, suggesting a sustainable approach to waste management and mitigation of environmental pollution. The addition of DRB resulted in the highest cumulative methane yield of 156 mL/(g·VS), representing a 37.1% enhancement compared to that in the control group. This improvement is attributed to the superior electron-transport capacity and abundant oxygen-containing functional groups of the co-pyrolytic biochar additive. In the DRB group, *Fastidiosipila* exhibited the highest relative abundance, while *Methanosarcina* reached a relative abundance of 54.2%, notably higher than the 47.3% observed in the control group. This finding underscores DRB’s role in enriching *Methanosarcina* populations and fostering interactions with *Fastidiosipila*. These interactions likely facilitated DIET mechanisms, which further accelerated methane production. Considering the promise of co-pyrolytic biochar additives, future research should prioritize their application and optimization at the pilot scale to advance practical implementation.

## Figures and Tables

**Table 1:** Physicochemical properties of materials.

	C (%)	N (%)	H (%)	S (%)	TS (%)	VS (%)	S_BET_ (m^2^/g)	Fe (mg/g)	Ni (mg/g)	Co (mg/g)
FW	0.7 ± 0.04	0.84 ± 0.06	12.34 ± 0.15	<0.1	23.70 ± 0.01	21.15 ± 0.26				
AS	9.98 ± 0.98	1.22 ± 0.09	5.73 ± 0.08	0.16 ± 0.01	2.29 ± 0.11	1.90 ± 0.33				
RS	34.68 ± 0.08	0.46 ± 0.01	4.93 ± 0.09	0.14 ± 0.01	92.56 ± 0.03	85.67 ± 0.26				
DG	20.80 ± 0.92	3.78 ± 0.09	3.27 ± 0.32	0.51 ± 0.02	95.62 ± 0.31	90.26 ± 0.55				
DB	13.87 ± 0.01	0.84 ± 0.04	0.95 ± 0.01	0.93 ± 0.01			21.91	3.57	0.008	0.754
RB	40.36 ± 0.03	0.55 ± 0.04	1.51 ± 0.02	0.25 ± 0.01			205.10	3.12	0.008	0.752
DRB	25.81 ± 0.20	1.19 ± 0.04	1.18 ± 0.03	0.36 ± 0.02			126.04	3.89	0.009	0.713

Note: FW: food waste; AS: anaerobic sludge; RS: rice straw; DG: digestate; DB: digestate biochar; RB: rice straw biochar; DRB: digestate- and rice-straw-derived co-pyrolysis biochar; TS: total solids; VS: volatile solids; S_BET_: specific surface area. Data for each group were obtained from three parallel tests.

## Data Availability

All data generated or analyzed during this study are included in this manuscript.

## Supplementary Materials

The following supporting information can be downloaded at: https://www.mdpi.com/article/10.3390/molecules30081766/s1, Figure S1: SEM images of RB, DB, and DRB; Figure S2: Material characteristic analysis: The XPS spectrum of (a) proportional distribution and (d,e) DB, (f,g) RB and (h,i) DRB; FTIR spectra (b); thermal stability (c); Figure S3: (a) N_2_ adsorption–desorption isotherms of DRB and (b) corresponding pore-size distribution curve; Figure S4: Analysis of bacterial communities at the OTU level during fermentation on (a) Day 5 and (b) Day 16; Analysis of archaea communities at the OTU level during fermentation on (c) Day 5 and (d) Day 16; Table S1: Proximate analysis and particle size of BC. Table S2: Total methane and biogas production of different groups.

## Abbreviations

The following abbreviations are used in this manuscript:

AD	anaerobic digestion
FW	food waste
DRB	digestate and rice straw
DIET	direct interspecies electron transfer
VFAs	volatile fatty acids
TVFAs	total volatile fatty acids
BC	biochar
TS	total solids
VS	volatile solids
DB	digestate biochar
RB	rice-straw biochar
AS	anaerobic sludge
DG	digestate
S_BET_	specific surface area
SEM	scanning electron microscope
FTIR	Fourier-transform infrared spectroscopy
BET	Brunauer–Emmett–Teller
COD	chemical oxygen demand
NH_3_-N	ammonia nitrogen
