# Supplementary material for: Enhancing Methane Production in Anaerobic Digestion of Food Waste Using Co-Pyrolysis Biochar Derived from Digestate and Rice Straw
Source: Molecules. 2025 Apr 15;30(8):1766. doi: 10.3390/molecules30081766 (PMC12029908; doi:10.3390/molecules30081766)
Supplement: Supplementary file 1 [file molecules-30-01766-s001.zip › molecules-3539372-supplementary.pdf]

Supplementary materials

Enhancing Methane Production in Anaerobic Digestion of Food Waste Using Co-Pyrolysis Biochar Derived from Digestate and Rice Straw

Qinyan Yang [1], Huanran Liu [1], Li Liu [1], Zhen Yan [2], Chunmeng Chui [3], Niannian Yang [3], Chen Wang [1], Guoqing Shen [1,4,*] and Qincheng Chen [1,*]

[1] School of Agriculture and Biology, Shanghai Jiao Tong University, Shanghai 200240, China
[2] Shanghai Pudong Development (Group) Co, Ltd., Shanghai 200127, China
[3] Shanghai Liming Resources Reuse Co, Ltd., Shanghai 201209, China
[4] Shanghai Yangtze River Delta Eco-Environmental Change and Management Observation and Research Station (Shanghai Urban Ecosystem Research Station), Ministry of Science and Technology, National Forestry and Grassland Administration, 800 Dongchuan Rd., Shanghai 200240, China
* Correspondence: gqsh@sjtu.edu.cn (G.S.); chenqincheng@sjtu.edu.cn (Q.C.)

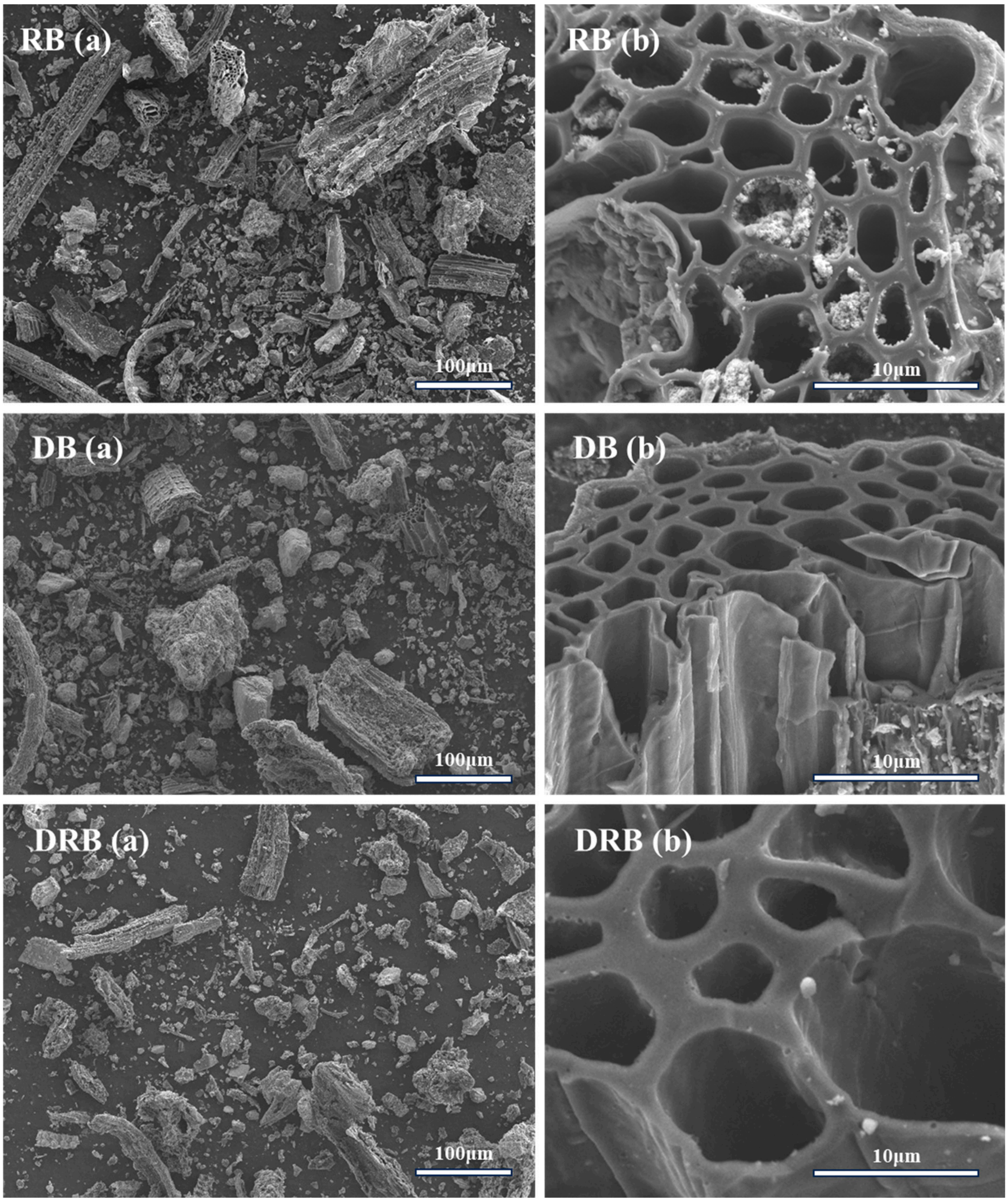

Figure S1 SEM images of RB, DB and DRB

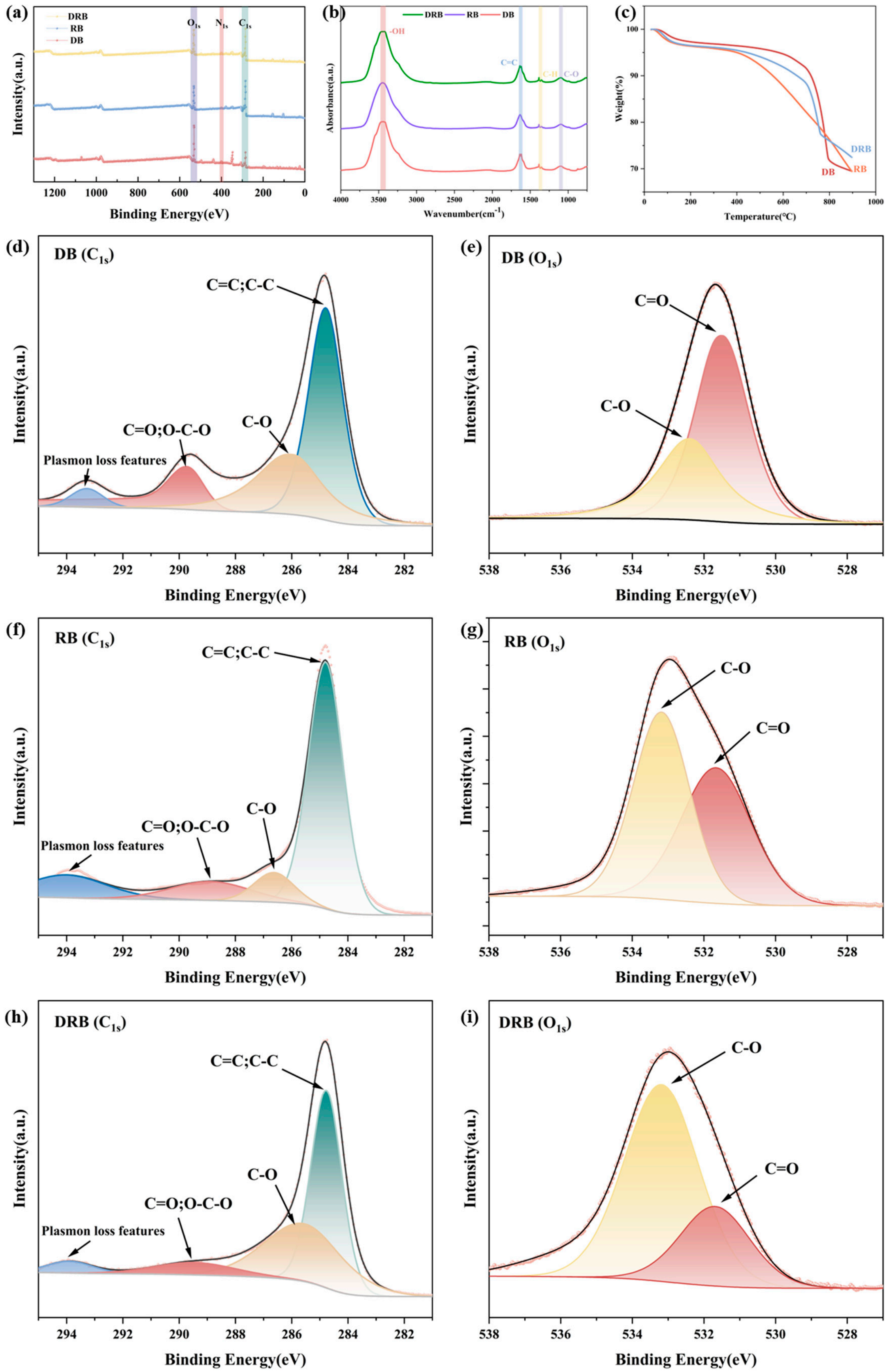

Figure S2 Material characteristic analysis: The XPS spectrum of (a) proportional distribution and (d,e) DB, (f,g) RB and (h,i) DRB; FTIR spectra (b); Thermal stability (c)

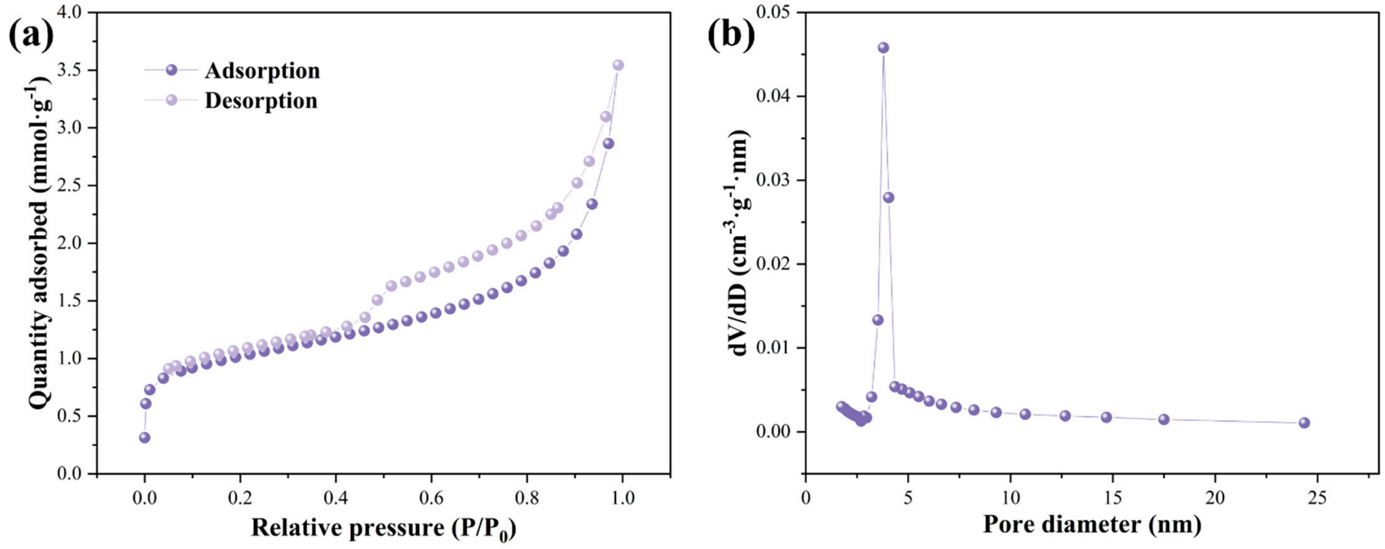

Figure S3 (a) N_2 adsorption-desorption isotherms of DRB and (b) the corresponding pore size distribution curve

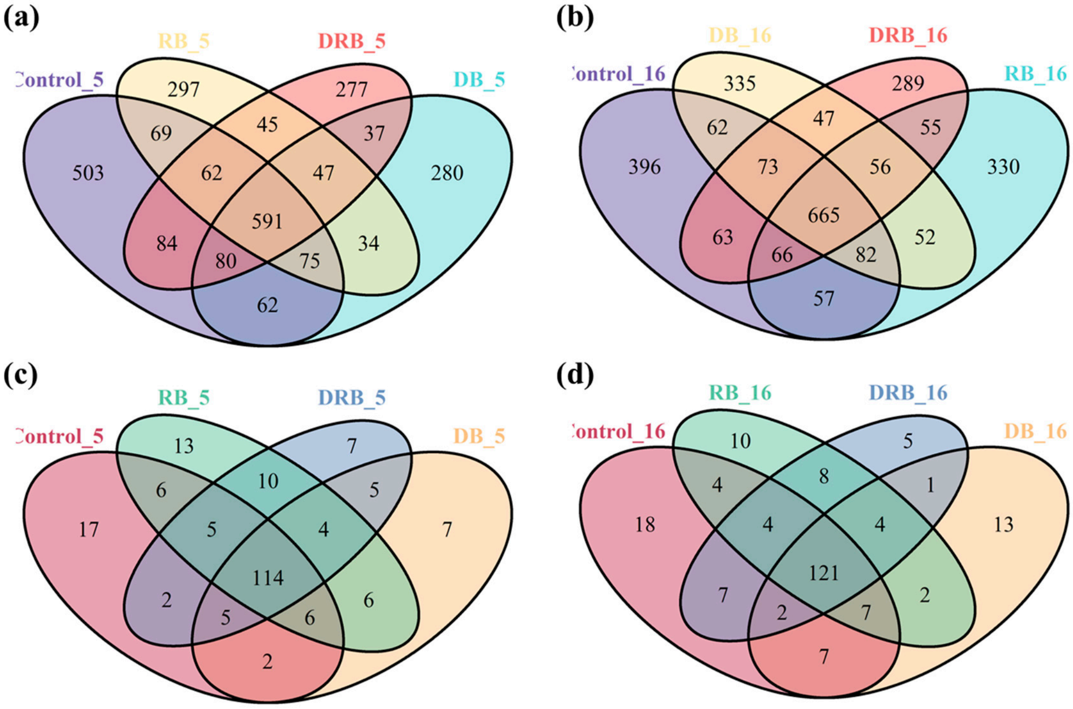

Figure S4 Analysis of bacterial communities at the OTUs level of fermentation on (a) Day 5 and (b) Day 16; Analysis of archaea communities at the OTU level of fermentation on (c) Day 5 and (d) Day 16

Table S1 Proximate analysis and particle size of BC.

Materials	Moisture (%)	Ash (%)	Particle size (mm)
DB	3.39±0.12	39.66±0.74	0.121-20.93
RB	2.55±0.03	18.55±1.04	0.138-5.714
DRB	2.07±0.05	18.02±0.54	0.126-3.111

Note: DB: digestate biochar; RB: rice straw biochar; DRB: digestate and rice straw co-pyrolysis biochar. Data for each group were obtained from three parallel tests.

Table S2 Total methane and biogas production of different groups.

Groups	Biogas (mL)	Methane (mL)
Control	1196.00 ±26.40	504.03 ±70.40
DB	1327.50 ±88.00	612.68 ±74.80
RB	1287.99 ±52.80	543.26 ±30.80
DRB	1375.00 ±35.20	696.86 ±22.00

Note: DB: digestate biochar; RB: rice straw biochar; DRB: digestate and rice straw co-pyrolysis biochar. Data for each group were obtained from three parallel tests.
